# The Influence of pH of Extracting Water on the Composition of Seaweed Extracts and Their Beneficial Properties on* Lepidium sativum*

**DOI:** 10.1155/2017/7248634

**Published:** 2017-04-05

**Authors:** Katarzyna Godlewska, Izabela Michalak, Łukasz Tuhy, Katarzyna Chojnacka

**Affiliations:** Department of Advanced Material Technologies, Faculty of Chemistry, Wrocław University of Science and Technology, Smoluchowskiego 25, 50-372 Wrocław, Poland

## Abstract

Baltic seaweeds were used to obtain aqueous extracts (E) through changing initial pH of deionised water added to algal biomass (EpH3·H_2_O, EpH7·H_2_O, and EpH10·H_2_O) and through changing pH of the mixture of algae and deionised water (EpH3, EpH7, and EpH10). Algal extracts were characterized in terms of the concentration of polyphenols and micro- and macroelements. The highest concentration of polyphenols was determined in extract EpH3 and the lowest in extract EpH10·H_2_O. It was found that the obtained extracts had similar concentrations of elements (except EpH3). The phytotoxicity of algal extracts (0.5, 2.5, and 10%) was examined in the germination tests on* Lepidium sativum*. No phytotoxic effects were observed. It was found that they had beneficial effects on the cultivated plants (length and weight). The best biostimulant effect was observed in the groups treated with EpH3 (2.5%), EpH7 (2.5%), and EpH7 (10%). The dry weight of plants was similar in all the groups. Algal extract also improved the multielemental composition of plant. The greatest concentration of total chlorophyll in plants was obtained by using extract EpH10·H_2_O, 0.5%. These results proved that algal extracts have high potential to be applied in cultivation of plants.

## 1. Introduction

Algae are mostly autotrophic organisms living in the aquatic or at least damp environment [1]. Their sizes range from a few micrometers (microalgae) up to several meters in case of macroalgae. Algae, due to the richness of organic and inorganic compounds, became the subject of many studies and were found to be useful in many industries, from cosmetics, through food to plant cultivation and animal breeding [2]. Among the most popular macroalgae, constituting a great source of biologically active compounds, green, red, and brown algae should be listed.

Bioactive compounds, naturally occurring in algae, were also found in extracts obtained from these organisms [3]. Algal extracts could be produced by either physical or chemical methods. Among all methods used for the algal extracts preparation (enzymatic extraction [4], microwave assisted extraction [5], and supercritical CO_2_ extraction [6]), extraction with the use of traditional solvents such as water, alcohol, inorganic acids, and bases seems to be the most popular. Algae extracts contain a large number of organic and mineral compounds (micro- and macronutrients). They are particularly rich in phytohormones (indoleacetic acids (IAA), commonly known as auxins, gibberellic acids, cytokinins, abscisic acids (ABA), and ethylene), complex organic compounds, vitamins, simple and complex sugars (polysaccharides like alginates, laminaria, and carrageenans), enzymes, N-containing compounds like betaines, proteins, and amino acids and sterols [7]. Among them, plant hormones—phytohormones—which are synthesized in the plant to regulate a multitude of essential cellular and tissue functions including stem elongation, root initiation, and tissue differentiation [8], play a significant role. The application of sequential extraction with solvent such as hot sodium oxalate, hot water, and KOH contributes to the extraction of biologically active compound (e.g., polysaccharides) from* Ulva rigida* [9]. Chemical components of algal extracts that affect plant growth were extensively described in many review papers [10, 11].

The positive effect of algal extracts on the plants growth and development was confirmed in many scientific publications. Increased chlorophyll content in tomatoes leaves and cucumbers, for both foliar and soil application was observed in comparison with control group. The main factor influencing the differences in chlorophyll content was high content of betaine in extracts from* Ascophyllum nodosum* [12]. It was also proved that the application of seaweed extracts in viticulture improved uptake of copper by plants [13]. The use of an extract from* Kappaphycus alvarezii* significantly improved the yield and nutrient uptake (primarily N, P, K, and S) for soybean [14] while the application of* Ascophyllum nodosum* extract in small concentrations had a positive effect on the radish development [15]. Algal extracts also improve resistance to stress factors, both biotic and abiotic [11].

In literature, a lot of attention is put on the description of the algal extracts impact on organic compounds composition, while there is little data including the elemental composition of preparations, which can be very important from the point of view of growing plants. The aim of the present work was to investigate the influence of different pH on chemical composition of seaweed extracts and to examine utilitarian properties of obtained preparations in laboratory tests on* Lepidium sativum*.

## 2. Materials and Methods

### 2.1. Chemicals

Sodium carbonate, ethanol, and methanol were purchased from POCH SA (Gliwice, Poland). Folin-Ciocalteu's phenol reagent, gallic acid, and nitric acid, 69%, were purchased from Merck KGaA (Darmstadt, Germany). All the reagents were of analytical grade.

### 2.2. Collection of Algae

The mixture of seaweeds,* Polysiphonia*,* Ulva*, and* Cladophora,* was collected directly from the water of Baltic Sea (Sopot, Poland) in August 2013. The biomass was rinsed with water in order to purify it from salt and partially from sand. In the next step, the impurities (e.g., shingles, sand, sea shells, and pieces of wood) were separated and the biomass was dried to 15% of moisture and ground to obtain particle size <0.3 mm (Wilk et al., 2014).

### 2.3. Extract Production

Extraction processes were made according to the modified procedures described by Sharma et al. [16]. To set appropriate pH (using pH meter Seven Multi; Mettler Toledo; Greifensee, Switzerland), hydrochloric acid (0.1 mol/L) and sodium hydroxide (0.1 mol/L) were used. In the first method, the dried and milled algal biomass (50 g) was added to 150 mL of deionised water with pH 3, 7, and 10, respectively (marked as extract (E) EpH3·H_2_O, EpH7·H_2_O, and EpH10·H_2_O). In the second method, 50 g of the dried and milled algal biomass was added to 150 mL of deionised water and then pH of the mixture was set to values 3, 7, and 10, respectively (marked as extract EpH3, EpH7, and EpH10). Then all flasks were shaken for 30 minutes, 150 rpm at 25°C (IKA KS 260 compact flat orbital shaker, Staufen, Germany). In the next step, each of six samples was centrifuged at 4250 rpm for 5 minutes (Heraeus Megafuge 40, rotor TX-750, Thermo Scientific, Waltham, MA, USA) and filtered using Whatman No. 1 filter paper. The obtained supernatant was taken as a 100% algal liquid extract.

### 2.4. Characteristics of Algal Extract

The characteristics of algal extracts were made according to the procedures described by Michalak et al. [17].

#### 2.4.1. Multielemental Composition

The content of elements in seaweeds, algal extracts, and cultivated plants was determined by ICP–OES iCAP 6500 Duo (Thermo Scientific, Waltham, MA, USA). The samples of seaweeds and garden cress (0.5 g) were purified from organic matter with nitric acid (69%; 5 mL) in Teflon bombs in a microwave oven Milestone Start D (Milestone S.r.l., Sorisole, Italy) and diluted with redemineralized water (Millipore Simplicity, Darmstadt, Germany) to 50 g. The samples were analyzed in three repetitions [17].

#### 2.4.2. Phenolic Compounds in the Algal Extracts

The concentration of phenolic compounds in obtained 100% algal extracts was determined according to the modified procedure described by Sim et al. [18]. The calibration curve was made with concentrations of gallic acid ranging from 25 to 1000 mg/L. Each concentration of gallic acid (0.1 mL) was mixed with deionised water (7.9 mL) and subsequently the Folin-Ciocalteu's phenol reagent (0.5 mL) was added to the samples. After 3 minutes, 1.5 mL of saturated sodium carbonate solution was added to the mixture and afterwards all mixtures were incubated for 30 minutes at 40°C. The blank contained only methanol. The absorbance (765 nm) was measured using a spectrophotometer, Varian Cary 50 Conc. Instrument (Victoria, Australia). The gallic acid calibration plot was obtained by plotting the absorbance versus the gallic acid concentration (mg/L) [17, 18].

### 2.5. Utilitarian Properties of Algal Extracts

#### 2.5.1. Germination Tests: Petri Dish Tests

The phytotoxicity of the algal extracts was evaluated in the germination tests on garden cress* (Lepidium sativum)*. The effect of different concentrations (0.5, 2.5, and 10%) of extracts on the growth of plants was tested. The dilutions were determined in previous work [19]. Experiments were conducted on Petri dishes, in 3 replicates for each group in standardized conditions using Jacobsen apparatus (Laborset, Lodz, Poland) according to the international norm (International Rules for Seed Testing, 2011—International Seed Testing Association (Bassersdorf, Switzerland)). On each Petri dish (diameter 85 mm), 50 seeds were placed on the universal soil (15 g) from “Lasland©” (Lasland sp. z o.o. Grądy, Poland). After stratification (5°C, 3 days), each dish was watered with algal extract (5 mL), whereas control group (C) was watered with distilled water (5 mL). After three days, all dishes were treated with the same doses of extracts or water. After seven days, cultivated plants were collected. Measurements of shoot length were carried out. The plant biomass was dried at temperature of 50°C (dryer Wamed SUP-30, Warsaw, Poland) and weighed (results expressed as a dry weight (d.w.)) [17].

#### 2.5.2. Chlorophyll Concentration in Extract from Garden Cress

The total chlorophyll (Total Chl), chlorophyll *a* (Chl(*a*)), and chlorophyll *b* (Chl(*b*)) in the fresh aerial parts of cultivated garden cress were determined by UV-VIS spectrophotometer (Varian Cary 50 Conc. Instrument, Victoria, Australia) at the following wavelengths: *λ* = 663 and 645 nm. Extractions were made in acetone [17, 20].

### 2.6. Statistical Analysis

The results were elaborated statistically by* Statistica *ver. 12 (StatSoft Polska Sp. z o.o., Kraków, Poland). Normality of distribution of experimental results was assessed by Shapiro-Wilk test. For normal distribution, homogeneity of variance was checked by means of the Brown-Forsythe test. For more than two groups, the differences were investigated with the (RIR) Tukey test, which compares all pairs of means following one-way ANOVA. Results were considered significantly different when *p* < 0.05. If the distribution of the results was other than normal, the Kruskal-Wallis test was used.

## 3. Results and Discussion

### 3.1. Characteristics of the Algal Extract

#### 3.1.1. Multielemental Composition of Algal Extracts

The appropriate fertilization enables obtaining quantitative and qualitative crops. Plants require at least 14 mineral elements, which include macroelements (nitrogen, phosphorus, potassium, calcium, magnesium, and sulphur), but also essential microelements (boron, iron, manganese, copper, zinc, nickel, and molybdenum). Deficiency of any of these mineral elements reduces plant growth and crop yields [21–23]. Therefore, algal extracts were examined whether they could be a source of valuable micro- and macroelements for plants. In Table 1, the elemental composition of the produced algal extracts is presented. It could be noticed that toxic elements were extracted from the raw algal biomass in low amounts in all examined variants. Extracts obtained through the changing of the value of water pH were similar in terms of the elemental composition. The highest extraction of elements was found to be for EpH7·H_2_O. It contained the highest concentration of Ca, Mg, Na, and S among macroelements and Cu, Mn, and Zn among microelements. The greatest concentration of P was noticed in EpH3·H_2_O. From this group, extraction in the group pH10·H_2_O appeared to be the least effective. In the case of the second extraction method it could be seen that low pH of water solution promoted the extraction of elements from the algal biomass. EpH3, in comparison with the other extracts, was especially rich in Ca, Mg, Na, and P and B, Fe, Mn, Ni, Si, and Zn. An extract which can be recommended for further study is EpH3. Table 1 shows also the elemental characteristics of algal extracts obtained by other extraction techniques using water as a solvent. The multielemental composition of extracts obtained by Microwave-Assisted Extraction (MAE) proved that temperature played a significant role in the extraction of elements; at lower temperature, lower amounts of elements were extracted. The extract obtained at 60°C was the most favorable in contrast to these produced at 25 and 40°C; the concentration of Fe and Si was four times higher and Zn two times higher than in the extract obtained at 25°C [17]. The extract obtained by boiling of algal biomass in water was especially rich in P, S, and B; on the other hand the extract obtained by soaking in water contained a great concentration of Ca, Mg, and Fe [19]. These essential mineral elements could be supplied to crops with algal extracts to achieve greater yields and also to increase their content in edible parts.

#### 3.1.2. Polyphenols in Algal Extracts

**Figure 1:**
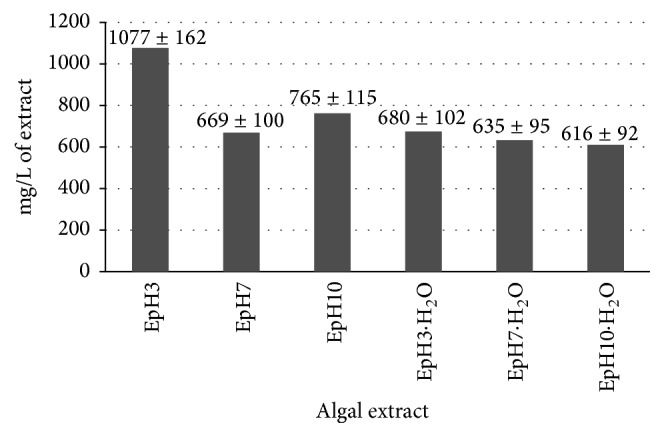
Total phenolic concentration (*N* = 3) in the obtained algal extracts.

Polyphenols are prevalent class of plant secondary metabolites and have received increasing attention in recent years due to their bioactive functions. These compounds may play different roles in human life and plant biology, such as antioxidant and protective agents against UV light, contributors to the taste of food, drink, and pharmaceuticals, defensiveness against herbivores and pathogens, and contributors to plant pigmentation but also as phytoalexins, antifeedants, and attractants for pollinators [24, 25]. Most of polyphenols present in plants are soluble in polar solvents and the extraction yields depend on extraction conditions. The low pH value of the extraction solution can prevent the oxidation of phenolics [26].  Figure 1 presents the total phenolic concentration [mg/L of gallic acid equivalents (GAEs) calculated from the equation: *y* = 0.0003 · *x* (*R*^2^ = 0.9959), where *y*  is absorbance and *x*  is concentration] in algal extracts. Generally, obtained extracts were similar in terms of the total phenolic concentration except EpH3 which was characterized by the highest concentration of these compounds (1077 mg/L). The results obtained by Horincar et al. [27] proved that water was the best solution to extract polyphenols in contrast to acetone, methanol, ethanol, and hexane. Water extract of* Cladophora vagabunda *contained 212 mg equivalent GAE/100 g of dry alga powder when methanolic extract contained 110 mg/100 g. In case of* Enteromorpha intestinalis, *its water extract had 146 mg/100 g while methanolic extract was only 75.5 mg/100 g [27]. Contrary data were presented by Narasimhan et al. [28] who determined polyphenol content in green seaweed samples,* Enteromorpha antenna* and* Enteromorpha linza,* extracted with different solvents such as chloroform, ethyl acetate, acetone, butanol, methanol, ethanol, and water. The total phenolic content was the highest for methanolic extract of* Enteromorpha antenna* (1.82 ± 0.05 GAE mg/g) and* E. linza* (0.912 ± 0.032 GAE mg/g). Ganesan et al. [29] prepared seaweed extracts by adding 20 g of algal sample (*Enteromorpha compressa*,* E. linza,* and* E. tubulosa*) to 200 mL of individual solvent (ethyl acetate, methanol, propanol, acetone, and water) for 6 h at room temperature using a Soxhlet extractor. The maximum total phenol content (%) was observed in the extract of acetone (11.63 ± 0.39), methanol (3.45 ± 0.18), and acetone (6.30 ± 0.06) for* E. compressa*,* E. linza,* and* E. tubulosa,* respectively. The lowest total phenol content was observed in the water extract of* E. compressa* (2.98 ± 0.39) and* E. linza* (1.33 ± 0.08), while ethyl acetate extract revealed the lowest phenols content in* E. tubulosa* (1.21 ± 0.05) [29]. Cho et al. [30] in their study used 50 g of milled sample of* Enteromorpha prolifera* and 500 mL of 95% ethanol to prepare algal extracts. Then the crude extract was dissolved in distilled water and then partitioned sequentially in three different solvents, namely,* n*-hexane, chloroform, and ethyl acetate. The total phenolic contents of the crude extract and solvent-partitioned fractions ranged from 46.2 to 80.4 mg GAE/g [30]. The variation of total phenol content between the* Chlorophyta* species might be due to extrinsic factors (herbivory pressure, irradiance, depth, salinity, nutrients, etc.) and intrinsic agents (type, age, and reproductive stage) [29].

### 3.2. Utilitarian Properties of Algal Extracts

Side effects of synthetic fertilizers on the environment stimulated the use of new natural sources of biostimulants and soil conditioners [31]. The application of seaweed extracts in plant cultivation can improve plant growth, yield, nutrient uptake, and resistance to biotic and abiotic stress and enhance postharvest shelf-life of perishable products [11, 31, 32]. They show activity at low concentrations (1 : 1000 dilution) [11]. Algal compounds affect cellular metabolism in treated plants due to the presence of macro- and microelement nutrients, amino acids, vitamins, cytokinins, auxins and abscisic acid- (ABA-) like growth substances. Seaweed products are easy to apply and relatively cheap (every year about 15 million metric tonnes are generated) [31].

#### 3.2.1. Total Height of the Cultivated Garden Cress

The height of plants (20 plants from each repetition,  *N* = 3) was determined for all the obtained extracts tested in three dilutions (0.5, 2.5, and 10%). Algal products showed varying degree of stimulant effect on the plants growth (Table 2). The control group was treated with water (pH 7.39). Generally, the plant height increased with increasing concentration of extracts. The best results were observed in the groups treated with extracts obtained by changing pH of the mixture of algae and water. The application of EpH3 2.5%, EpH7 10%, and EpH7 2.5% influenced the plants height, which was 26.5, 25.4, and 25.0% longer than in the control group, respectively. The use of extracts obtained by changing pH of water resulted in the lowest biostimulant effect. In the groups EpH7·H_2_O 0.5% > EpH10·H_2_O 0.5% > EpH7·H_2_O 2.5% > EpH7·H_2_O 10% plants were shorter than in the control group, respectively, by 9.3 > 7.6 > 1.3 > 0.93%. In order to verify the statistically significant differences (for *p* < 0.05) between the tested groups, two analyses, using STATISTICA software, were performed. In the first comparison, between the control group and groups treated with extracts obtained by changing pH of mixture of water and algae, the statistically significant differences were found between the control and all other groups and between EpH3 2.5% and EpH10 0.5%. The data distribution was nonnormal; therefore the Kruskal-Wallis test was chosen. In the second comparison, between the control group and preparations received by changing pH of water used for the extraction process, the statistically significant differences were found between EpH3·H_2_O 2.5% and EpH7·H_2_O 0.5%, EpH7·H_2_O 0.5% and EpH3·H_2_O 10%, EpH10·H_2_O 2.5% and EpH7·H_2_O 0.5%, EpH10·H_2_O 10% and EpH7·H_2_O 0.5%, and EpH10·H_2_O 10% and EpH10·H_2_O 0.5%. The distributions were normal; therefore Tukey test was used.

In the literature, several methods are used for the production of aqueous algal extracts. In the work of Fakihi Kachkach et al. [33], extracts were produced from dried biomass of* Ulva rigida* that was heated with distilled water in a ratio of 10 : 100 (w/v) for 2 minutes. Different concentrations of extracts (0.5, 1, 2, and 4 mg of dried seaweeds per mL of distilled water) were investigated in terms of growth of garden cress. Results showed that all extracts significantly affected growth of roots and stems of* L. sativum. *The highest stimulant effect was observed in the group treated with 1 mg/mL, whereas the concentration 4 mg/mL showed lower stimulation of roots and stems growth. Latique et al. [34] investigated the effect of* Ulva lactuca *extract (obtained by boiling one kilogram of fresh seaweed with a liter of distilled water for one hour) on the growth of bean plants (*Phaseolus vulgaris *L.). Seaweed extracts were applied as a foliar spray at different concentrations: 6, 12.5, 25, 50, and 75%. Two concentrations, 25 and 50%, provided the significant effects on plant growth and the maximum effect was found in group treated with 25% extract (44.7% longer than in the control group without seaweed extract). In the paper of Osman and Salem [35], the effect of foliar applications of different concentrations of seaweed extract of* Ulva lactuca *on sunflower (*Helianthus annuus* L.) was presented. Authors applied two concentrations 0.4 and 0.6% w/v, 3 times: first one at the seedling stage (20 days after sowing), the second at the flowering stage (40 days after sowing), and the third one before yield stage (70 days after sowing). Seaweed extracts significantly increased plant height when compared to the control group without algal extract. The highest sunflower plants were recorded in the group treated with 0.4%* U. lactuca *(37.3% higher).

#### 3.2.2. Weight of the Cultivated Plants

The results of the research showed that the dry weight of* Lepidium sativum *was similar in all the groups, taking into account all extracts and dilutions (Table 3). It could be noticed that the highest dry weight was in the groups treated with EpH10 2.5% (16.4% heavier than in the control) and EpH7 2.5% (12.9% heavier). Extract obtained with water with pH 10 applied at a concentration 10% emerged to be the least effective (25.9% lighter). Osman and Salem [35] showed that very low concentration (0.4 and 0.6%) of* U. lactuca *extracts promoted dry weight of sunflower. In the group treated with extract at concentration 0.6%, plants were heavier by about 87.5% than in the control. In the work of Khairy et al. [36], the seaweed extracts were prepared by soaking overnight one kilogram of* Ulva lactuca *and* Enteromorpha compressa *in a litre of distilled water. The filtrate was used to prepare different concentrations of algal products (5%, 10%, and 15%). Four foliar applications of each concentration were applied at 30, 51, 72, and 93 days from sowing. The algal extracts stimulated the crop yield of broad bean* (Vicia faba) *as the total number of seeds and their weight per plot. Where the total number of seeds was 164 per plot for control, it was 266 (394.4 kg) by the application of 10% extract concentration of* E. intestinalis *and 267 (423.6 kg) for* U. lactuca.*

#### 3.2.3. Multielemental Composition of the Cultivated Garden Cress

Iron, zinc, selenium, calcium, magnesium, and copper deficiencies are common in many countries. The main causes of this situation are crop production in areas with low mineral phytoavailability and consumption of scarce amount of fish or animal products and crops with inherently low mineral contents [37]. Dietary diversification, supplementation, food fortification, and increasing mineral contents in edible crops could be used to combat dietary micronutrient deficiencies [37, 38]. Perhaps, biofortification of staple food crops is the most feasible approach to lower the number of severely malnourished people and help to maintain improved nutritional status [38–40]. The present study proved that the application of seaweed extracts can increase the content of micro- and macroelements in plants (Tables 4 and 5). Algal extract, obtained with water with pH 10, applied at the concentration 10% (pH10·H_2_O (10%)) affected to the highest extent the content of macroelements in the cultivated garden cress; for example, plants contained about 78.5% more Na, 36% more Mg, 35% more K, and 15% more Ca than plants in the control group. The highest amounts of P and S were in the group treated with pH10·H_2_O (0.5%) (23 and 39% more, resp.). In the case of microelements, the highest content of B was in the group treated with EpH3 10% (273% more), Cu in the group EpH10·H_2_O 0.5% (109% more), Si in EpH7·H_2_O 2.5% (153% more), Zn in EpH7 0.5% (97% more), Mn in EpH3·H_2_O 2.5% (76% more), and Mo in groups EpH3·H_2_O 0.5% and EpH3·H_2_O 2.5% (94%) compared to the control group. The cultivated* Lepidium sativum *contained mainly these elements, which occurred in the largest concentrations in the algal extract.

#### 3.2.4. Chlorophyll Concentration in the Extract from Cultivated Cress

The concentration of total chlorophyll (Total Chl), Chl(*a*), and Chl(*b*) in extracts from the cultivated plants was determined from the following equations [20] and is presented in Table 6:

(1) Total Chl=8.02·A663+20.2·A645CChla=12.7·A663−2.69·A645CChlb=22.9·A645−4.68·A663.

In most cases, total chlorophyll concentration in extracts from* Lepidium sativum *in the experimental groups was higher than in the control group. Only in the group treated with EpH7 2.5% the concentration of chlorophyll *b* was 3.3% lower than in the control group. The highest concentration of chlorophyll *a*, chlorophyll *b*, and total chlorophyll in plant was in the group treated with EpH10·H_2_O 0.5% (65.8%, 70.3%, and 67.3% more than in the control group, resp.). These results proved that algal extracts increased plant productivity, resulting in increased chlorophyll content. Latique et al. [34] also reported that higher chlorophyll *a* content was found for 25%* Ulva rigida *extract (20.08 mg/g d.w.) when compared with the control plants (4.4 mg/g d.w.). Osman and Salem [35] observed that foliar application of aqueous extract of* Ulva lactuca* significantly increased the chlorophyll content in sunflower. In the first stage (45 days from plantation), the highest chl(*a*) was detected in sunflower plant treated with 0.4%* U. lactuca *(7.40 mg/g), while the highest chl(*b*) was recorded in plant treated with 0.6%* U. lactuca *(3.36 mg/g). In the second stage (65 days from plantation), the highest chlorophyll *a* and chlorophyll *b* were detected in plant treated with 0.6%* U. lactuca *(6.28 and 2.61 mg/g, resp.). Our previous study [17, 19] also proved that aqueous seaweed extracts can enhance plant chlorophyll content.

## 4. Conclusions

*Polysiphonia*,* Ulva*, and* Cladophora* derived seaweed extracts may be beneficial in increasing the growth parameters (plant height and weight) and chlorophyll content and enhancing nutrient uptake by plants. The application of algal products may deliver substantial economic and environmental benefits. Research will be continued to launch the product in the market (field test, development of the production technology, proposal of an installation, preliminary economic analysis, etc.).

## Figures and Tables

**Table 1:** Multielemental composition of the obtained algal extracts (*N* = 3): comparison with literature data.

Element	Baltic seaweeds, (mg/kg dry weight)	EpH3	EpH7	EpH10	EpH3·H_2_O	EpH7·H_2_O	EpH10·H_2_O	Extract obtained by boiling in water	Extract obtained by soaking in water	Extract obtained by MAE 25°C	Extract obtained by MAE 40°C	Extract obtained by MAE 60°C
	Godlewska et al., 2016	Present work						Godlewska et al., 2016		Michalak et al., 2015		
		mg/L										
Macroelements												
Ca	40292 ± 8058	1553 ± 311	275 ± 41	160 ± 24	266 ± 40	305 ± 46	285 ± 43	333 ± 50	410 ± 61	354 ± 53	363 ± 54	365 ± 54
K	5082 ± 1016	1068 ± 214	1236 ± 247	2264 ± 453	1069 ± 214	1027 ± 205	1023 ± 205	969 ± 145	978 ± 147	868 ± 130	901 ± 135	951 ± 142
Mg	3181 ± 636	606 ± 91	277 ± 42	140 ± 21	285 ± 43	308 ± 46	291 ± 44	300 ± 45	357 ± 54	303 ± 45	311 ± 46	322 ± 48
Na	6354 ± 1271	1418 ± 284	1190 ± 238	1123 ± 225	1168 ± 234	1286 ± 257	1237 ± 248	1239 ± 248	1302 ± 260	1050 ± 211	1200 ± 240	1250 ± 250
P	1155 ± 231	4422 ± 884	40.61 ± 6.09	33.660 ± 5.049	161 ± 24	27.290 ± 4.094	35.96 ± 5.39	34.7 ± 5.2	5.22 ± 0.78	9.52 ± 1.43	18.3 ± 2.7	32.9 ± 4.9
S	8614 ± 1723	541 ± 81	541 ± 81	596 ± 89	492 ± 74	547 ± 82	523 ± 79	670 ± 100	599 ± 90	562 ± 84	582 ± 87	702 ± 105

Microelements												
B	97.83 ± 14.68	17.78 ± 2.67	1.57 ± 0.24	0.380 ± 0.057	3.69 ± 0.55	2.86 ± 0.43	2.70 ± 0.41	6.50 ± 0.97	2.62 ± 0.39	3.3 ± 0.5	3.44 ± 0.51	4.74 ± 0.71
Cu	12.69 ± 1.90	0.07 ± 0.02	0.230 ± 0.035	0.27 ± 0.04	0.0300 ± 0.0075	0.310 ± 0.047	0.100 ± 0.015	0.140 ± 0.021	0.020 ± 0.005	0.1170 ± 0.0117	0.148 ± 0.022	0.108 ± 0.016
Fe	6660 ± 1332	26.99 ± 4.05	11.45 ± 1.72	19.48 ± 2.92	3.94 ± 0.59	6.11 ± 0.92	6.820 ± 1.023	2.53 ± 0.38	17.6 ± 2.6	1.19 ± 0.18	2.17 ± 0.28	4.47 ± 0.70
Mn	232 ± 35	14.60 ± 2.19	1.73 ± 0.26	0.78 ± 0.12	2.060 ± 0.309	2.32 ± 0.35	2.11 ± 0.32	2.43 ± 0.36	3.71 ± 0.56	2.52 ± 0.37	2.62 ± 0.39	3.07 ± 0.46
Mo	0.312 ± 0.047	0.003 ± 0.001	0.010 ± 0.003	0.0300 ± 0.0075	0.0100 ± 0.0025	0.0100 ± 0.0025	0.0100 ± 0.0025	0.020 ± 0.005	0.000	0.001 ± 0.000	0.179 ± 0.026	0.0108 ± 0.0027
Si	906.90 ± 136.04	27.05 ± 4.06	14.43 ± 2.17	16.54 ± 2.48	6.19 ± 0.93	9.64 ± 1.45	10.84 ± 1.63	9.10 ± 1.36	9.73 ± 1.46	3.10 ± 0.46	6.69 ± 1.00	11.9 ± 1.8
Zn	64.94 ± 9.74	3.41 ± 0.51	0.270 ± 0.041	0.54 ± 0.08	0.140 ± 0.021	0.210 ± 0.032	0.170 ± 0.026	0.240 ± 0.036	0.100 ± 0.015	0.0746 ± 0.0112	0.20 ± 0.03	0.169 ± 0.025

Toxic metals												
As	3.902 ± 0.507	0.26 ± 0.04	0.150 ± 0.023	0.180 ± 0.027	0.170 ± 0.026	0.150 ± 0.023	0.140 ± 0.021	0.170 ± 0.022	0.160 ± 0.021	0.246 ± 0.032	0.150 ± 0.019	0.198 ± 0.025
Cd	0.7067 ± 0.0919	0.010 ± 0.003	<LLD	<LLD	<LLD	<LLD	<LLD	<LLD	<LLD	<LLD	0.001 ± 0.000	0.001 ± 0.000
Pb	7.028 ± 0.914	0.030 ± 0.008	0.0300 ± 0.0075	0.0300 ± 0.0075	0.0100 ± 0.0025	0.020 ± 0.005	0.020 ± 0.005	0.040 ± 0.008	0.0100 ± 0.0025	0.0098 ± 0.0020	0.0104 ± 0.0021	0.032 ± 0.006

<LLD: below detection limit.

**Table 2:** Total height of the cultivated garden cress in the experimental groups.

Extract	Average height $\overset{-}{x}$ (cm) ± SD (*N* = 3)
Control	5.36 ± 0.59

EpH3 0.5%	6.45 ± 0.44
EpH3 2.5%	6.78 ± 0.40^a^
EpH3 10%	6.64 ± 0.51

EpH7 0.5%	6.35 ± 0.70
EpH7 2.5%	6.70 ± 0.67
EpH7 10%	6.72 ± 0.64

EpH10 0.5%	6.30 ± 0.61^a^
EpH10 2.5%	6.61 ± 0.52
EpH10 10%	6.67 ± 0.63

EpH3·H_2_O 0.5%	5.50 ± 0.76
EpH3·H_2_O 2.5%	5.57 ± 0.71^a^
EpH3·H_2_O 10%	5.56 ± 0.85^a^

EpH7·H_2_O 0.5%	4.86 ± 0.79^a^
EpH7·H_2_O 2.5%	5.29 ± 0.64
EpH7·H_2_O 10%	5.31 ± 1.03

EpH10·H_2_O 0.5%	4.95 ± 1.04^a^
EpH10·H_2_O 2.5%	5.63 ± 0.79^a^
EpH10·H_2_O 10%	5.64 ± 0.96^a^

^a^Statistically significant differences (*p* < 0.05).

**Table 3:** The dry weight of cultivated garden cress in the experimental groups.

Extract	Average dry weight $\overset{-}{x}$ (g) ± SD (*N* = 3)
Control	0.0739 ± 0.0033

EpH3 0.5%	0.0773 ± 0.0015
EpH3 2.5%	0.0788 ± 0.0050
EpH3 10%	0.0800 ± 0.0031

EpH7 0.5%	0.0778 ± 0.0030
EpH7 2.5%	0.0834 ± 0.0010
EpH7 10%	0.0778 ± 0.0015

EpH10 0.5%	0.0782 ± 0.0054
EpH10 2.5%	0.0860 ± 0.0009
EpH10 10%	0.0826 ± 0.0040

EpH3·H_2_O 0.5%	0.0679 ± 0.0028
EpH3·H_2_O 2.5%	0.0665 ± 0.0012
EpH3·H_2_O 10%	0.0697 ± 0.0015

EpH7·H_2_O 0.5%	0.0615 ± 0.0009
EpH7·H_2_O 2.5%	0.0606 ± 0.0016
EpH7·H_2_O 10%	0.0612 ± 0.0005

EpH10·H_2_O 0.5%	0.0554 ± 0.0006
EpH10·H_2_O 2.5%	0.0596 ± 0.0023
EpH10·H_2_O 10%	0.0547 ± 0.0129

**Table 4:** Multielemental composition of cultivated garden cress (mg/kg d.w.) treated with EpH3, EpH7, and EpH10 (*N* = 3).

Element	Control group	EpH3			EpH7			EpH10		
		0.5%	2.5%	10%	0.5%	2.5%	10%	0.5%	2.5%	10%
Macroelements										
Ca	11161 ± 394	10542 ± 2108	10928 ± 2186	11282 ± 2256	10338 ± 2068	11299 ± 2260	11404 ± 2281	11070 ± 2214	10597 ± 2119	10829 ± 2166
K	58067 ± 3802	65119 ± 13024	65322 ± 13064	69453 ± 13890	66512 ± 13302	67006 ± 13401	68669 ± 13734	65273 ± 13055	64931 ± 12986	69337 ± 13867
Mg	6974 ± 68	7284 ± 1457	7437 ± 1487	7642 ± 1528	7629 ± 1526	7280 ± 1456	7173 ± 1435	7046 ± 1409	7031 ± 1406	6935 ± 1387
Na	1850 ± 8	2132 ± 426	2133 ± 427	2950 ± 590	2180 ± 436	2541 ± 508	2849 ± 570	2794 ± 559	3067 ± 613	3272 ± 654
P	16475 ± 964	18119 ± 3624	18099 ± 3620	18092 ± 3618	17604 ± 3521	17486 ± 3497	15965 ± 3193	16902 ± 3380	16830 ± 3366	15169 ± 3034
S	15508 ± 939	16966 ± 3393	16423 ± 3285	13042 ± 2608	18165 ± 3633	16662 ± 3332	14214 ± 2843	17158 ± 3432	16282 ± 3256	13280 ± 2656

Microelements										
B	7.30 ± 0.82	16.89 ± 2.53	19.94 ± 2.99	27.19 ± 4.08	15.85 ± 2.38	18.30 ± 2.75	16.23 ± 2.44	13.79 ± 2.07	15.29 ± 2.29	17.74 ± 2.66
Cu	4.24 ± 0.07	6.72 ± 1.01	5.59 ± 0.84	6.82 ± 1.02	7.75 ± 1.16	6.04 ± 0.91	5.25 ± 0.79	8.50 ± 1.28	5.65 ± 0.85	5.10 ± 0.77
Fe	278 ± 11	221 ± 33	212 ± 32	209 ± 31	215 ± 32	245 ± 37	244 ± 37	262 ± 39	255 ± 38	259 ± 39
Mn	41.88 ± 1.85	72.19 ± 10.83	75.65 ± 11.35	75.71 ± 11.36	71.39 ± 10.71	71.39 ± 10.71	69.00 ± 10.35	70.18 ± 10.53	69.87 ± 10.48	66.97 ± 10.05
Mo	1.20 ± 0.01	2.14 ± 0.32	2.21 ± 0.33	2.05 ± 0.31	1.76 ± 0.26	2.0 ± 0.3	1.93 ± 0.29	2.07 ± 0.31	2.15 ± 0.32	2.01 ± 0.30
Si	182 ± 22	265 ± 40	228 ± 34	297 ± 45	260.90 ± 39.14	252 ± 38	243 ± 37	436.4 ± 65.5	275 ± 41	262.1 ± 39.3
Zn	58.22 ± 0.71	91.35 ± 13.70	86.77 ± 13.02	83.43 ± 12.52	114.9 ± 17.2	83.51 ± 12.53	79.07 ± 11.86	86.83 ± 13.03	79.52 ± 11.93	76.81 ± 11.52

Toxic metals										
As	0.35 ± 0.12	0.36 ± 0.05	0.58 ± 0.09	0.86 ± 0.13	0.700 ± 0.105	0.60 ± 0.09	0.660 ± 0.099	0.71 ± 0.11	0.59 ± 0.09	0.61 ± 0.09
Cd	0.26 ± 0.04	0.35 ± 0.05	0.32 ± 0.05	0.28 ± 0.04	0.2937 ± 0.0441	0.340 ± 0.051	0.31 ± 0.05	0.35 ± 0.05	0.32 ± 0.05	0.30 ± 0.05
Pb	1.19 ± 0.01	1.33 ± 0.20	1.16 ± 0.17	1.44 ± 0.22	1.104 ± 0.166	1.19 ± 0.18	1.33 ± 0.19	1.64 ± 0.25	1.58 ± 0.24	1.40 ± 0.21

**Table 5:** Multielemental composition of cultivated garden cress (mg/kg d.w.) treated with EpH3·H_2_O, EpH7·H_2_O, and EpH10·H_2_O.

Element	Control group	EpH3·H_2_O			EpH7·H_2_O			EpH10·H_2_O		
		0.5%	2.5%	10%	0.5%	2.5%	10%	0.5%	2.5%	10%
Macroelements										
Ca	11161 ± 39	8462 ± 1692	9381 ± 1876	10405 ± 208	9289 ± 1858	10156 ± 2031	11640 ± 2328	10175 ± 203	10576 ± 211	12893 ± 257
K	58067 ± 3802	61096 ± 12219	69817 ± 13963	59688 ± 11938	59637 ± 11927	64736 ± 12947	64666 ± 12933	65172 ± 13034	66197 ± 13239	78177 ± 15635
Mg	6974 ± 68	7660 ± 1532	7491 ± 1498	7331 ± 1466	8054 ± 1611	7872 ± 1574	8008 ± 1602	8581 ± 1716	8683 ± 1737	9494 ± 1899
Na	1850 ± 8	1299 ± 260	1631 ± 326	2515 ± 503	1314 ± 263	1682 ± 336	2701 ± 540	1561 ± 312	1729 ± 346	3302 ± 660
P	16475 ± 96	18449 ± 369	18375 ± 367	15947 ± 318	18413 ± 3683	17941 ± 3588	16560 ± 3312	20254 ± 405	19298 ± 386	16608 ± 332
S	15508 ± 93	18283 ± 365	18686 ± 373	13808 ± 276	18142 ± 3628	17498 ± 3500	14471 ± 2894	21623 ± 432	18200 ± 364	16684 ± 333

Microelements										
B	7.30 ± 0.82	10.71 ± 1.61	11.79 ± 1.77	12.90 ± 1.94	8.88 ± 1.33	10.12 ± 1.52	11.24 ± 1.69	9.76 ± 1.46	10.10 ± 1.52	11.84 ± 1.78
Cu	4.24 ± 0.07	5.105 ± 0.76	4.119 ± 0.61	5.11 ± 0.77	4.49 ± 0.67	3.492 ± 0.524	3.632 ± 0.545	8.88 ± 1.33	6.49 ± 0.97	5.56 ± 0.83
Fe	278 ± 11	146 ± 22	163 ± 25	141 ± 21	164 ± 25	139 ± 21	134 ± 20	172 ± 26	179 ± 27	139 ± 21
Mn	41.88 ± 1.8	75.31 ± 11.3	78.40 ± 11.7	68.50 ± 10.2	75.07 ± 11.26	69.91 ± 10.49	44.74 ± 6.71	52.38 ± 7.86	48.28 ± 7.24	49.00 ± 7.35
Mo	1.20 ± 0.01	2.33 ± 0.35	2.33 ± 0.35	2.08 ± 0.31	2.07 ± 0.31	1.971 ± 0.296	1.249 ± 0.187	1.04 ± 0.16	1.31 ± 0.20	1.292 ± 0.19
Si	182 ± 22	174 ± 26	256 ± 38	203 ± 31	203 ± 31	460 ± 69	276 ± 41	142 ± 21	325 ± 49	135 ± 20
Zn	58.22 ± 0.7	85.93 ± 12.8	89.07 ± 13.3	104 ± 16	104 ± 16	95.96 ± 14.39	69.53 ± 10.43	94.24 ± 14.1	85.29 ± 12.7	69.89 ± 10.4

Toxic metals										
As	0.35 ± 0.12	0.27 ± 0.04	0.500 ± 0.07	0.76 ± 0.11	0.51 ± 0.08	0.8361 ± 0.12	0.63 ± 0.10	0.53 ± 0.08	0.32 ± 0.05	0.64 ± 0.10
Cd	0.26 ± 0.04	0.30 ± 0.05	0.26 ± 0.04	0.31 ± 0.05	0.27 ± 0.04	0.2528 ± 0.03	0.15 ± 0.02	0.26 ± 0.04	0.23 ± 0.04	0.25 ± 0.04
Pb	1.19 ± 0.01	0.68 ± 0.10	0.61 ± 0.09	0.58 ± 0.09	0.6026 ± 0.09	0.77 ± 0.12	0.6593 ± 0.098	0.5059 ± 0.0	0.52 ± 0.08	0.553 ± 0.08

**Table 6:** Chlorophyll concentration in the extracts of cultivated garden cress (mg/L) (*N* = 3).

Sample	Concentration of chlorophyll *a*	Concentration of chlorophyll *b*	Total chlorophyll concentration
Control	15.53 ± 2.33	5.95 ± 0.89	21.48 ± 3.22
EpH3 0.5%	19.35 ± 2.90	6.79 ± 1.02	26.14 ± 3.92
EpH3 2.5%	20.26 ± 3.04	6.28 ± 0.94	26.54 ± 3.98
EpH3 10%	17.89 ± 2.68	6.11 ± 0.92	24.00 ± 3.6
EpH7 0.5%	18.92 ± 2.84	6.69 ± 1.00	25.60 ± 3.84
EpH7 2.5%	16.62 ± 2.49	5.76 ± 0.86	22.37 ± 3.36
EpH7 10%	22.79 ± 3.42	8.64 ± 1.30	31.43 ± 4.71
EpH10 0.5%	20.29 ± 3.04	7.12 ± 1.07	27.41 ± 4.11
EpH10 2.5%	17.81 ± 2.67	5.98 ± 0.90	23.78 ± 3.57
EpH10 10%	19.08 ± 2.86	6.62 ± 0.99	25.69 ± 3.85
EpH3·H_2_O 0.5%	21.24 ± 3.19	7.90 ± 1.19	29.14 ± 4.37
EpH3·H_2_O 2.5%	21.78 ± 3.27	8.84 ± 1.33	30.62 ± 4.59
EpH3·H_2_O 10%	22.76 ± 3.41	8.50 ± 1.28	31.26 ± 4.69
EpH7·H_2_O 0.5%	23.49 ± 3.52	9.04 ± 1.36	32.52 ± 4.88
EpH7·H_2_O 2.5%	23.38 ± 3.51	8.31 ± 1.25	31.68 ± 4.75
EpH7·H_2_O 10%	19.95 ± 2.99	7.09 ± 1.06	27.03 ± 4.06
EpH10·H_2_O 0.5%	25.74 ± 3.86	10.13 ± 1.52	35.87 ± 5.38
EpH10·H_2_O 2.5%	25.71 ± 3.86	8.84 ± 1.33	34.54 ± 5.18
EpH10·H_2_O 10%	22.04 ± 3.31	7.65 ± 1.15	29.69 ± 4.00
